# Long-Term Outcomes of Primary Trabeculectomy in Diabetic Patients without Retinopathy with Primary Angle-Closure Glaucoma

**DOI:** 10.1155/2017/7947854

**Published:** 2017-02-26

**Authors:** Jian Liu, Miaomiao Zhang, Bin Li, Jianrong Wang

**Affiliations:** Department of Ophthalmology, The Second People's Hospital of Jinan, No. 148 Jingyi Road, Jinan 250001, China

## Abstract

*Purpose*. To evaluate primary trabeculectomy with adjunctive mitomycin-C (MMC) in diabetic patients without retinopathy with primary angle-closure glaucoma (PACG). *Design*. This is a retrospective case series comparison. *Participants*. This retrospective trial compared outcomes of 88 eyes that underwent trabeculectomy in patients with diabetes mellitus (DM) without retinopathy and in 97 patients without DM. *Methods*. In this study, the intraocular pressure (IOP), visual acuity, visual field, and postoperative complications were compared between the two groups. Qualified surgical success is defined as an IOP between 6 and 18 mmHg with or without topical antiglaucoma medication. *Results*. After a follow-up of 5 years, the IOP decreased from a mean basal IOP of 27.8 ± 7.3 mmHg to 15.0 ± 5.6 mmHg in the DM group and from 27.3 ± 6.0 mmHg to 12.4 ± 5.3 mmHg in the control group. The mean number of antiglaucoma medications was 3.4 ± 1.3 and 3.3 ± 1.2 preoperatively (*P* = 0.587) whereas it was 1.7 ± 1.5 and 1.1 ± 1.4 at the 5-year follow-up (*P* = 0.049). The 5-year qualified surgical success rates were 42.9% and 65.4% for both groups (*P* = 0.046; log-rank test). Encysted blebs were seen in 21 (23.9%) patients in the DM group and in 12 (12.4%) patients in the control group (*P* = 0.041). *Conclusion*. PACG patients with DM without retinopathy undergoing primary trabeculectomy with MMC may have a lower long-term surgical survival rate compared with patients without DM.

## 1. Introduction

According to current literature, diabetes mellitus (DM) is a risk factor for the failure of trabeculectomy [1–3]. However, published data were mainly focused on trabeculectomy in diabetic eyes with proliferative disease or with different stages of diabetic retinopathy [4–6]. Law et al. reported the long-term outcomes of primary trabeculectomy with mitomycin-C (MMC) in diabetic patients without retinopathy with primary open-angle glaucoma (POAG) [7]. The result indicated that POAG patients with DM without retinopathy undergoing primary trabeculectomy may have a lower long-term surgical survival rate compared with patients without DM. There is a paucity of long-term follow-up data in the literature on the effects of trabeculectomy with MMC on diabetic patients without retinopathy with primary angle-closure glaucoma (PACG) [8]. It is unknown whether the outcomes of primary trabeculectomy with MMC in PACG patients with controlled DM are comparable with similar surgery in PACG patients without DM.

The purpose of this study is to evaluate the long-term outcomes of primary trabeculectomy with MMC in diabetic patients without retinopathy with PACG.

## 2. Materials and Methods

This is a retrospective case series comparison of the outcomes of primary trabeculectomy with MMC between PACG patients with DM (DM group) and PACG patients without DM (control group). A total of 88 eyes from 88 patients with DM who had undergone primary trabeculectomy with MMC were compared with 97 eyes from 97 patients without DM. The study was approved by The Second People's Hospital of Jinan and followed the tenets of the Declaration of Helsinki. Medical records of patients with PACG who had undergone trabeculectomy with MMC between July 2005 and December 2008 and had at least 1 year of follow-up were reviewed.

PACG was defined as the structural alteration of the optic disc typical for glaucomatous optic neuropathy or visual field defect typical for glaucoma, in the presence of a closed anterior chamber angle on gonioscopy (closed anterior chamber angle was defined as an occludable angle with appositional closure for 180 degrees or more, along with some combinations of the presence of peripheral anterior synechiae). Exclusion criteria included glaucoma subtypes or optic neuropathy other than PACG, diabetic or nondiabetic retinal pathology based on documented dilated fundus examination within 6 months, history of retinal photocoagulation, previous intraocular procedure other than cataract surgery, and concurrent procedures performed with trabeculectomy.

Patients are grouped by the presence or absence of DM. The DM group was composed of controlled diabetic patients without retinopathy. The control group was selected from all nondiabetic PACG patients undergoing MMC trabeculectomy. If the patient had two eyes that would qualify, the first operated eye was elected. Biomicroscopic, gonioscopic, and fundoscopic examinations were performed in all patients before the surgery. Data collected included demographic data (age and gender); medical data (fasting blood serum sugar level collected in the preoperative physical examination within 1 month of surgery and haemoglobin A1c (HgbA1c) level whenever available); preoperative and postoperative ocular data (intraocular pressure (IOP), best-corrected visual acuity (BCVA; logMAR), glaucoma medication, slit-lamp examination of the anterior segment, fundus examination, lens status, and visual field result whenever available); and surgical data (intraoperative and postoperative complications and any surgeries or procedures performed after trabeculectomy). Data for visual acuity, IOP, and antiglaucoma medication were collected preoperatively and at intervals of 6 months (±2 weeks) and each year (±1 months) thereafter after trabeculectomy. IOP was defined as the average of three IOP measurements by Goldmann tonometry. Snellen visual acuity was converted to logMAR scale for comparison. LogMAR values of 1.40, 2.70, and 3.70 were assigned to counting fingers, hand motions, and light perception, respectively [9]. Only reliable visual field results (24–2 Swedish interactive threshold algorithm—standard algorithm) were included (reliability was defined as fixation loss of <20% and false positive and negative rate of <33%). Postoperative data were collected for all consecutive visits.

All surgeries have been performed by the same single surgeon. After the local anesthesia, the bulbus was stabilized with an atraumatic 4-0 silk traction suture suspending the superior rectus muscle. Trabeculectomy was performed with a fornix-based conjunctival flap (approximately between 11 and 1 o'clock). Then, a scleral flap (rectangular, half-thickness, and 4 × 5 mm in size) was dissected until the entire corneoscleral limbus was exposed. A surgical sponge (Medtronic Xomed, Inc.) measuring 6 mm × 6 mm which was soaked with 0.4 mg/ml MMC (mitomycin-C powder was reconstituted in sterile water to give the final concentration) was placed under the sclera flap for 2 min [10, 11]. After the MMC application area was fully irrigated with balanced salt solution, trabeculectomy (approximately 1 × 3 mm) was performed parallel to the limbus with a peripheral iridectomy. The scleral flap was sutured with 10/0 nylon sutures (two at the apex). The conjunctiva was continuously sutured with a 10/0 nylon suture. Postoperatively, patients were instructed to stop their preoperative glaucoma medications and to use tobramycin 0.3% 4 times daily for 2 weeks and prednisolone acetate 1% eye drops every 2 hours while awake for 1 week followed by 4 times daily for 4 weeks and then tapered off over 3 to 4 weeks. Atropine 1% twice daily was added when the anterior chamber shallowed or when IOP was <6 mmHg.

Complete success was defined as the IOP between 6 and 18 mmHg without medications. Qualified surgical success was defined as the IOP between 6 and 18 mmHg regardless of topical antiglaucoma medication. A failure is defined as the IOP ≥ 18 mmHg regardless of any medication. Hypotony was diagnosed in cases with IOP levels of <6 mmHg. Needling of encysted blebs was not considered a failure. The conjunctival leakage was observed under cobalt blue slit-lamp illumination after a moistened sterile fluorescein strip was applied on the bleb surface. A leak was diagnosed if there was any apparent aqueous stream from any leaking point.

Data are presented as mean ± SD. All statistical analyses were performed using SPSS statistical software (ver. 18.0; SPSS, Inc., Chicago, IL, USA). Independent *t*-tests were used to evaluate the between-group and within-group differences, respectively. Categorical variables and proportions were analyzed using Pearson's *χ*^2^ tests. Success rates in both groups were compared using the Kaplan-Meier life table analysis and the log-rank test. Statistical significance was defined as a *P* value of <0.05.

## 3. Result

A total of 88 eyes (88 patients) with type 2 DM that had undergone primary trabeculectomy with MMC were compared with 97 eyes (97 patients) without DM. All recruited patients were Asians. Table 1 summarises the baseline and intraoperative characteristics of the two groups. As expected, the DM group had a higher preoperative fasting blood serum sugar level than the control group (128.6 ± 21.3 and 94.9 ± 16.1, resp., *P* < 0.001). Since patients with DM were either on diet control (12 eyes, 13.6%) or diabetic medical therapy (76 eyes, 86.4%), their preoperative blood serum sugar levels were not very high. All cases in the DM group had a preoperative HgbA1c level checked in the preoperative physical examination (mean: 6.8 ± 0.8%). Overall, 69 eyes (78.4%) in the DM group and 75 eyes (77.3%) in the control group had reliable preoperative visual field results for comparison. The differences in MD (mean deviation; dB) on the visual field between the two groups were not statistically significant (*P* = 0.586).

After a follow-up of 5 years, the IOP decreased from a mean basal IOP of 27.8 ± 7.3 mmHg to 15.0 ± 5.6 mmHg in the DM group and from 27.3 ± 6.0 mmHg to 12.4 ± 5.3 mmHg in the control group. Mean postoperative IOP of the control group was lower than that of the DM group at all visit intervals starting at 1 year after the trabeculectomy, and the differences were statistically significant at the 2-, 3-, 4-, and 5-year follow-ups (Table 2). The mean number of antiglaucoma medications was 3.4 ± 1.3 and 3.3 ± 1.2 preoperatively (*P* = 0.587) whereas it was 1.7 ± 1.5 and 1.1 ± 1.4 at 5-year follow-up (*P* = 0.049). Mean postoperative number of antiglaucoma medications in the control group was also lower than that in the DM group at all visit intervals 1 year after the trabeculectomy (Table 2).

In the DM group, the mean BCVA at baseline was 0.28 ± 0.31 logMAR and 0.51 ± 0.21 logMAR at the 5-year follow-up. Differences between the preoperative and postoperative BCVA were statistically significant (*P* = 0.000, Table 2). In the control group, the mean BCVA at baseline was 0.28 ± 0.33 logMAR, and at the 5-year follow-up, it was 0.50 ± 0.15 units. The reduction of vision at the 5-year follow-up was also statistically significant (*P* = 0.001). BCVA differences between the groups were not statistically significant at any visit interval.

After a follow-up of 5 years, 37 eyes (42.0%) in the DM group and 41 eyes (42.3%) in the control group had reliable preoperative visual field results. At the 5-year follow-up, the MD was −15.2 ± 10.2 dB in the DM group and −14.9 ± 9.8 dB in the control group. There were no statistically significant differences between the preoperative and the postoperative visual field results in either group (*P* = 0.251 and *P* = 0.113, resp.). MD differences between the groups were not statistically significant at any visit interval (Table 3).

**Figure 1:**
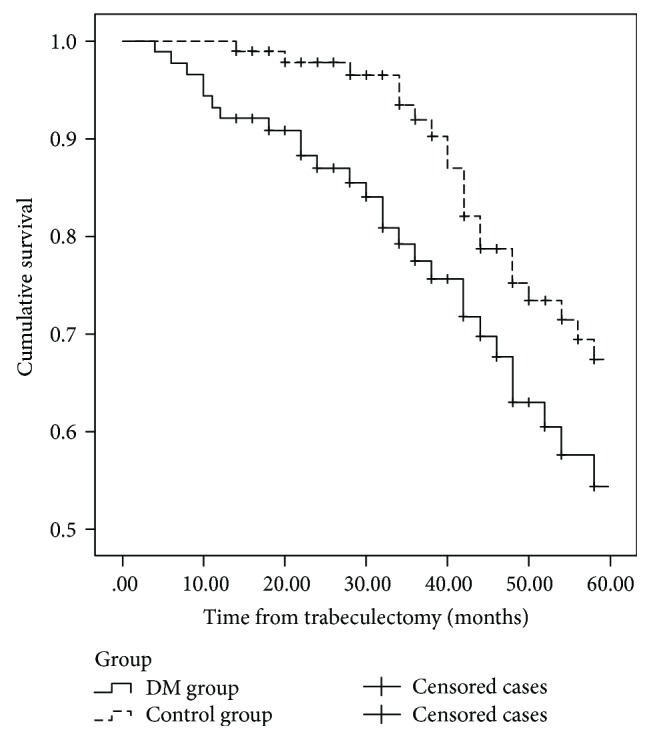
Survival curve with completed success defined as IOP between 6 and 18 mmHg without antiglaucoma medication. At the 5-year follow-up, the rates of complete surgical success were 35.7% (15 eyes) in the DM group and 59.6% (31 eyes) in the control group (*P* = 0.038; log-rank test).

**Figure 2:**
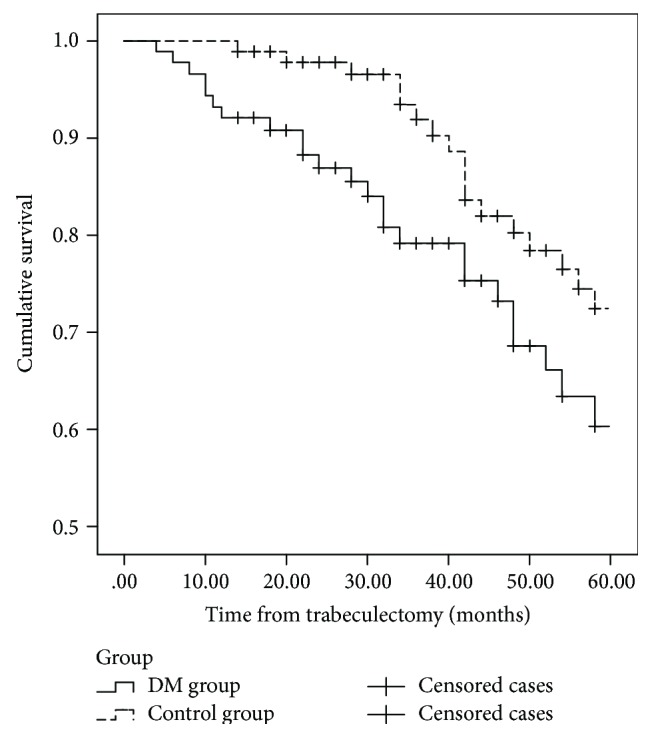
Survival curve with qualified success defined as IOP between 6 and 18 mmHg with or without topical antiglaucoma medication. At the 5-year follow-up, the rates of qualified success were 42.9% (18 eyes) in the DM group and 65.4% (34 eyes) in the control group (*P* = 0.046; log-rank test).

The rates of complete surgical success were 35.7% (15 eyes) in the DM group and 59.6% (31 eyes) in the control group (*P* = 0.038; log-rank test) (Figure 1), and the rates of qualified success were 42.9% (18 eyes) in the DM group and 65.4% (34 eyes) in the control group (*P* = 0.046; log-rank test) (Figure 2) at the 5-year follow-up.

There were 2 patients with persistent choroidal detachment in the control group which was resolved with surgical intervention (subretinal fluid drainage). Anterior chamber reformation was performed in all cases with prolonged postoperative hypotony, which is always accompanied with prolonged shallow anterior chamber. All eyes with an encysted bleb underwent 1-time needling procedure without injecting any antifibrotic agent (*P* = 0.041). There were 2 (2.3%) cases of endophthalmitis in the DM group. After vitrectomy, both patients lost light perception. There was no other patient who lost light perception in both groups (Table 4).

## 4. Discussion

As far as we know, there is no report about the effect of trabeculectomy on diabetic PACG patients without retinopathy. There are few studies examining the effect of trabeculectomy on diabetic eyes with DM, but the results are conflicting [1–2, 12–14]. Some studies reported that DM was associated with poorer outcome in trabeculectomy [1, 2, 12, 13]. However, the Fluorouracil Filtering Surgery Study showed that diabetic status was not associated with poorer outcome in trabeculectomy [14].

To our knowledge, this is the first study to investigate the outcome of trabeculectomy with MMC in diabetic PACG patients without retinopathy. The results showed that long-term IOP was less controlled in the eyes of diabetic PACG patients without retinopathy than in the eyes of those without DM. Mean postoperative IOP of the DM group was higher than that of the control group. The rates of qualified success were 42.9% in the DM group and 65.4% in the control group (*P* = 0.046; log-rank test) at the 5-year follow-up. Mean postoperative IOP was 12.4 mmHg in the DM group and 15.0 mmHg in the control group (*P* = 0.024). The majority of trabeculectomy failures are characterized by a marked inflammatory response in the conjunctival dermis and Tenon's capsule [15]. An excessive breakdown in the blood aqueous barrier following cataract surgery has been linked to diabetes, as an increased incidence of postoperative inflammation was assessed clinically [16, 17]. It could be expected that similar effects occur in diabetic patients following trabeculectomy, and this may be the explanation for our findings.

The results demonstrated that there were no statistically significant differences in the rate of complications such as hypotony, choroidal detachment, shallow anterior chamber, and conjunctival leakage between the two groups. The incidence of encysted bleb ranged from 2.5% to 29% in literature [18]. In the present study, more encysted blebs in the DM group (23.9%) than in the control group (12.4%) were observed. A plausible biologic explanation of this association is that the diabetic patients have an altered inflammatory response that may augment scarring after trabeculectomy [12]. The augment of scarring might contribute to the formation of high IOP and encysted blebs. There were 2 cases of endophthalmitis in the DM group. Fortunately, there were no cases of endophthalmitis in the control group. We assumed that it is related to the higher susceptibility to bacterial infections in the DM group [19].

There was a mild decrease of vision in the DM group and in the control group, and the decrease achieved statistical significance. Matlach et al. have reported a slight decrease in visual acuity after 12 months compared to baseline values [20]. In a similar study, researchers reported that they have not found any changes in visual acuity after surgery [21]. In the recent report of treatment outcomes after 5 years of follow-up of the Tube Versus Trabeculectomy Study [22], which is a multicentre randomized clinical trial comparing tube shunt procedure versus trabeculectomy in patients who had previous trabeculectomy or cataract surgery, a much greater decrease of visual acuity was reported in the trabeculectomy group (from baseline of 0.37 logMAR units or 20/50 Snellen equivalent to 0.71 logMAR units or 20/100 Snellen equivalent at 5 years). In our cases, we recorded that there were complications affecting visual acuity such as hypotony and cataract formation. Decrease of visual acuity over the long-term follow-up in glaucoma patients after glaucoma surgery is common and can be a result of many causes including progression of glaucoma, complications of surgery, worsening of cataract, or development of other ocular disorders.

At the 5-year follow-up, there was a mild decrease of MD in both groups, but the decrease did not achieve statistical significance. The differences in MD between the two groups were not statistically significant at the last visit. Previous studies reported that lowering IOP slowed the advancement of visual field damage in glaucoma patients [23–25]. However, even if the IOP can be substantially lowered, the reduction of mean and peak IOPs does not always prevent visual field progression [26]. Few reports have appeared in the literature regarding long-term IOP control and long-term visual field progression after trabeculectomy [27, 28]. Hong et al. believed that long-term IOP fluctuation may play a significant role in visual field deterioration in glaucoma patients who always keep low IOPs [28].

While the postoperative IOP, number of medications required, and surgical success were all worse in the DM group, the postoperative vision and visual field mean deviation were similar between the two groups. There are two reasons: first, the retrospective comparative nature of this study may have caused a bias. Second, a small number of included patients will affect the final statistical results.

This study has some limitations. First, the retrospective comparative nature of the study may have caused a bias. Second, we were unable to assess the severity of the glaucoma by the visual field defect, although this has been suggested to play little part in the outcome of trabeculectomy surgery [29]. A prospective randomized controlled trial should be conducted in the future to clarify our results.

In conclusion, diabetic PACG patients without retinopathy undergoing primary trabeculectomy with MMC may not achieve the same long-term IOP control that nondiabetic patients achieve, despite the use of MMC and the same surgical technique, and in the absence of diabetic retinopathy and with blood sugars under medical or diet control. However, further prospective studies are expected.

## Figures and Tables

**Table 1:** Demographic and intraoperative characteristics of the DM group and the control group.

	DM group (*n* = 88)	Control group (*n* = 97)	*P*
Age (±SD, year)	51.8 ± 10.07	53.9 ± 8.67	0.111^∗^
Female/male	52/36	60/37	0.701^#^
Phakia/pseudophakia	80/8	86/11	0.473^#^
Blood sugar level (mg/dl)	128.6 ± 21.3	94.9 ± 16.1	0.000^∗^
Preoperative IOP (mmHg)	27.8 ± 7.3 (22–50)	27.3 ± 6.0 (22–44)	0.610^∗^
Preoperative number of antiglaucoma medications	3.4 ± 1.3	3.3 ± 1.2	0.587^∗^
BCVA (logMAR)	0.28 ± 0.31	0.28 ± 0.33	1.000^∗^
Humphrey visual fields			
MD^&^, mean ± SD (dB)	−12.8 ± 10.2	−11.9 ± 9.6	0.586^∗^

^∗^Independent *t*-test.

^#^Pearson's *χ*^2^ test.

^&^Mean deviation on visual field.

**Table 2:** Intraocular pressure, medical therapy, and BCVA at follow-up.

Postoperative follow-ups	DM group	Control group	*P*
*6 months*			
Number of eyes	88	97	
IOP (mmHg)	12.0 ± 4.6	11.2 ± 4.6	0.239
Glaucoma medications	0.8 ± 0.9	0.6 ± 0.9	0.198
Visual acuity in logMAR	0.29 ± 0.32	0.29 ± 0.33	1.000

*1 year*			
Number of eyes	88	97	
IOP (mmHg)	12.9 ± 4.9	11.4 ± 4.5	0.036
Glaucoma medications	1.3 ± 1.3	0.6 ± 0.9	0.000
BCVA (logMAR)	0.33 ± 0.42	0.32 ± 0.45	0.876

*2 years*			
Number of eyes	76	81	
IOP (mmHg)	13.3 ± 5.2	11.6 ± 4.9	0.036
Glaucoma medications	1.3 ± 1.3	0.8 ± 1.2	0.013
BCVA (logMAR)	0.36 ± 0.46	0.34 ± 0.49	0.793

*3 years*			
Number of eyes	60	63	
IOP (mmHg)	13.5 ± 5.6	11.6 ± 5.0	0.049
Glaucoma medications	1.3 ± 1.4	0.8 ± 1.3	0.042
BCVA (logMAR)	0.42 ± 0.59	0.40 ± 0.61	0.853

*4 years*			
Number of eyes	50	59	
IOP (mmHg)	13.9 ± 4.8	12.0 ± 4.9	0.044
Glaucoma medications	1.5 ± 1.5	1.0 ± 1.1	0.047
BCVA (logMAR)	0.47 ± 0.43	0.45 ± 0.43	0.809

*5 years*			
Number of eyes	42	51	
IOP (mmHg)	15.0 ± 5.6	12.4 ± 5.3	0.024
Glaucoma medications	1.7 ± 1.5	1.1 ± 1.4	0.049
BCVA (logMAR)	0.51 ± 0.21	0.50 ± 0.15	0.789

**Table 3:** Mean deviation on visual field in patients at follow-up (dB).

Postoperative follow-ups	DM group (*n*)	Control group (*n*)	*P*
1 year	−12.1 ± 9.5 (57)	−11.4 ± 9.4 (56)	0.695
2 years	−13.7 ± 9.9 (47)	−12.8 ± 9.5 (57)	0.638
3 years	−14.4 ± 9.7 (41)	−13.1 ± 10.0 (48)	0.537
4 years	−14.8 ± 9.6 (40)	−14.5 ± 9.7 (44)	0.887
5 years	−15.2 ± 10.2 (37)	−14.9 ± 9.8 (41)	0.895

**Table 4:** Postoperative complications and postsurgical interventions (%).

	DM group	Control group	*P*
Prolonged postoperative hypotony (≥1 month)	2 (2.2)	4 (4.1)	0.478
Choroidal detachment	6 (6.8)	9 (9.3)	0.540
Conjunctival leakage	6 (6.8)	6 (6.2)	0.861
Shallow anterior chamber	10 (11.4)	12 (12.4)	0.833
Encysted blebs	21 (23.9)	12 (12.4)	0.041
Endophthalmitis	2 (2.3)	0	0.135
Postsurgical interventions			
Conjunctival suture	3 (3.4)	3 (3.1)	0.903
Bleb needling	21 (23.9)	12 (12.4)	0.041
Anterior chamber reformation	2 (2.3)	4 (4.1)	0.478
Subretinal fluid drainage	0	2 (2.1)	0.176
Repeat trabeculectomy	8 (9.1)	9 (9.3)	0.965
Cataract surgery	8 (9.1)	6 (6.2)	0.456
